# Resident macrophage subpopulations occupy distinct microenvironments in the kidney

**DOI:** 10.1172/jci.insight.161078

**Published:** 2022-10-24

**Authors:** Matthew D. Cheung, Elise N. Erman, Kyle H. Moore, Jeremie M.P. Lever, Zhang Li, Jennifer R. LaFontaine, Gelare Ghajar-Rahimi, Shanrun Liu, Zhengqin Yang, Rafay Karim, Bradley K. Yoder, Anupam Agarwal, James F. George

**Affiliations:** 1 Department of Surgery,; 2 Department of Nephrology Research and Training Center,; 3 Department of Cellular Developmental and Integrative Biology,; 4 Department of Medicine, and; 5 Department of Veterans Affairs, University of Alabama at Birmingham, Birmingham, Alabama, USA.

**Keywords:** Immunology, Nephrology, Bioinformatics, Innate immunity, Macrophages

## Abstract

The kidney contains a population of resident macrophages from birth that expands as it grows and forms a contiguous network throughout the tissue. Kidney-resident macrophages (KRMs) are important in homeostasis and the response to acute kidney injury. While the kidney contains many microenvironments, it is unknown whether KRMs are a heterogeneous population differentiated by function and location. We combined single-cell RNA-Seq (scRNA-Seq), spatial transcriptomics, flow cytometry, and immunofluorescence imaging to localize, characterize, and validate KRM populations during quiescence and following 19 minutes of bilateral ischemic kidney injury. scRNA-Seq and spatial transcriptomics revealed 7 distinct KRM subpopulations, which are organized into zones corresponding to regions of the nephron. Each subpopulation was identifiable by a unique transcriptomic signature, suggesting distinct functions. Specific protein markers were identified for 2 clusters, allowing analysis by flow cytometry or immunofluorescence imaging. Following injury, the original localization of each subpopulation was lost, either from changing locations or transcriptomic signatures. The original spatial distribution of KRMs was not fully restored for at least 28 days after injury. The change in KRM localization confirmed a long-hypothesized dysregulation of the local immune system following acute injury and may explain the increased risk for chronic kidney disease.

## Graphical abstract

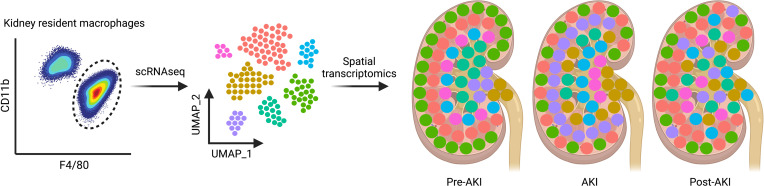

## Introduction

Macrophages are ubiquitous in the kidney (1). They perform numerous functions within the kidney, including integration of signals related to tissue health, phagocytosis of debris and apoptotic cells, defense against infectious agents entering via the urinary tract, and response to physical injury or damage by pharmacologic agents. In a normal murine kidney, macrophages constitute approximately 50%–60% of CD45^+^ immune cells, and of those, the majority are tissue resident, having seeded into the kidney in embryonic and early life from the fetal yolk sac, fetal liver, and bone marrow (2). Kidney-resident macrophages (KRMs) are defined as such because they are replenished primarily through self-renewal and receive no further input from the peripheral circulation (3, 4). The KRM population can be distinguished from infiltrating macrophages by their high expression of F4/80 (also known as Emr1 and Ly71) and intermediate expression of CD11b (integrin α_m_β_2_, also known as Mac-1) (5). Infiltrating macrophages, which are exclusively bone marrow derived, turnover within 2 weeks (3) and express lower levels of the F4/80 antigen and higher levels of CD11b (F4/80^int^, CD11b^hi^). Parabiosis studies following an ischemic event show that these characteristics do not change after kidney injury (3).

Kidney macrophages are known to be important in acute kidney injury (AKI), but their conflicting roles in the pathogenesis of and recovery from injury are poorly understood. In models of AKI, such as kidney ischemia/reperfusion injury in mice, the extent and timing of macrophage depletion affect the outcome of injury (6, 7).

KRMs are distributed throughout the kidney, which contains distinct physiological microenvironments along the nephron. Macrophage location has been shown to affect function in other organs/tissues (8–12). Therefore, KRMs may have specialized functions tailored to the local cellular environment and may possess transcriptional programs to respond distinctly to acute injury. We have previously shown in an ischemia/reperfusion injury model that there are changes in the expression of selected surface molecules, such as MHC II, and in transcriptional programming, as measured by bulk mRNA sequencing (3). It is not clear if the observed transcriptomic and phenotypic shifts reflect alterations in global macrophage programming or previously indistinguishable subpopulations.

We used single-cell RNA-Seq (scRNA-Seq) and spatial transcriptomics to demonstrate that KRMs consist of at least 7 major subpopulations localized to defined zones within the kidney. The distinct transcriptomic programs of each subpopulation suggest specialized functions, including type I IFN responses, heme/iron metabolism, inflammation, or antibacterial responses. Following acute ischemic injury, the transcriptomic programming is altered, and the delineation of the subpopulations within specific zones is lost and does not return to normal by 28 days after injury, possibly reflecting chronic immune dysregulation.

## Results

### Generation of a comprehensive single-cell KRM data set.

**Figure 1:**
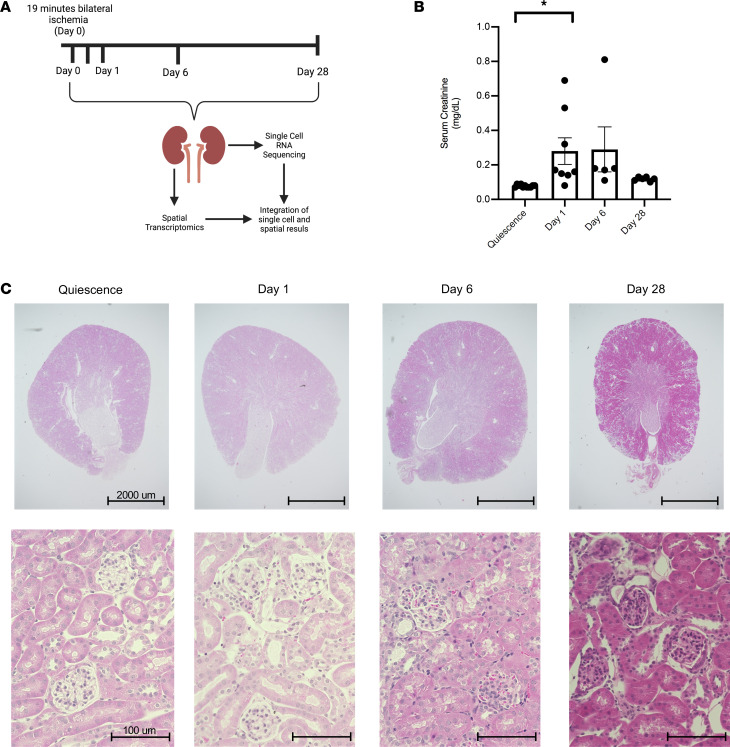
Model of acute kidney injury. (**A**) Schematic depicting work flow for scRNA-Seq and spatial transcriptomics. Mice were subjected to bilateral ischemia/reperfusion injury for 19 minutes. Kidneys were harvested at day 0, 12 hours, and days 1, 6, and 28 after injury. Kidneys were either utilized for spatial transcriptomics or digested and flow sorted for KRMs and subjected to scRNA-Seq. There were 3 biological replicates per time point. (**B**) Serum creatinine levels (mg/dL) at quiescence (day 0) and days 1, 6, and 28 after injury from at least 2 independent experiments. Data are reported as mean ± SEM. A 1-way ANOVA was used to determine the statistical significance between groups. *P < 0.05. (**C**) H&E-stained kidney sections at quiescence and days 1, 6, and 28 after injury. Scale bar: 2000 μm (top row); 100 μm (bottom row).

**Figure 2:**
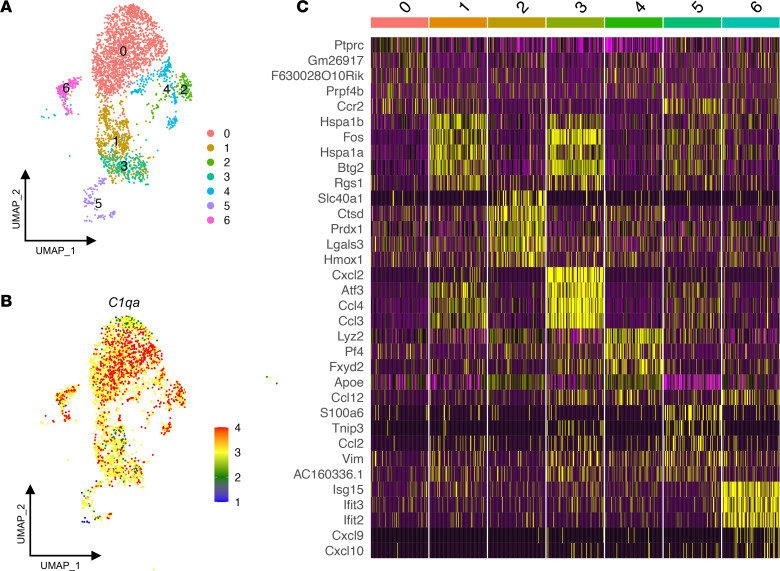
Single-cell RNA-Seq and spatial transcriptomics reveal distinct subpopulations of kidney-resident macrophages. (**A**) Uniform manifold approximation and projection (UMAP) plot of sequenced kidney-resident macrophages (KRMs) demonstrating 13 clusters. Contaminating kidney cells and clusters representing less than 1% were removed to leave 7 unique clusters in quiescence. (**B**) *C1qa* expression in all KRM clusters during quiescence. (**C**) Heatmap of top 5 differentially expressed genes among each subpopulation in quiescence ordered by adjusted *P* value.

We performed scRNA-Seq on CD45^+^TCRb^–^CD19^–^NK1.1^–^Gr-1^–^CD11b^int^F4/80^hi^ KRMs identified and sorted by flow cytometry as described previously (Supplemental Figure 1; supplemental material available online with this article; https://doi.org/10.1172/jci.insight.161078DS1) (3). The KRMs were obtained from the kidneys of quiescent (uninjured) mice and those subjected to 19 minutes of bilateral warm ischemia (37°C ± 1°C rectal temperature) under ketamine-xylazine anesthesia (Figure 1A). Histologic changes and serum creatinine confirmed the initial injury on day 1 after ischemia, with observed hypercellularity and an average increase of 0.21 mg/dL in serum creatinine and subsequent recovery of function from days 6 to 28 after injury (Figure 1, B and C). Samples from 3 quiescent mice and 3 mice each at 12 hours and days 1, 6, and 28 days after injury were sequenced using the 10X Genomics 3′ Chromium platform. In total, 58,304 KRMs were sequenced and retained based on the expression of *Adgre1* (F4/80), *Itgam* (CD11b), and C1qa (3, 13). The data from all time points were integrated using Harmony to facilitate a direct comparison of the analyses at each time point by minimizing batch effects and nonbiologic variation. After quality control, 3000 variable genes were identified for all groups, and after normalization and scaling, the cells were clustered. Dimensional reduction using uniform manifold approximation and projection (UMAP) resolved 13 clusters (Supplemental Figure 2A). Elimination of contaminating kidney parenchymal cells and clusters constituting equal to or less than 1% of the total number of cells left 7 clusters, indicating that the KRM population is composed of at least 7 major distinct subpopulations with unique transcriptomic signatures (Figure 2A and Supplemental Figure 2B). KRMs were distinguished from parenchymal cells by expression of canonical markers such as of C1qa (Figure 2B) (13).

### Delineation of transcriptionally distinct KRMs in quiescent mice.

We initially analyzed the quiescent samples to characterize the 7 major subpopulations in the uninjured state (Figure 2A). A heatmap of the top 5 significant differentially expressed genes (DEGs) in each cluster illustrates distinct transcriptional profiles and provides potential markers for identification (Figure 2C). Very few transcripts were uniformly expressed at high levels in cluster 0, although *Ccr2* and *Ptprc* (CD45) were among the DEGs that defined this cluster (Figure 2C). The top DEGs in the remaining 6 clusters indicated at least one specific function. Cluster 1 expressed heat shock protein transcripts and the *Fos* and *Btg2* transcription factor/cofactors, which are associated with the immediate early response (Figure 2C). These transcripts may be involved in homeostatic function or may indicate priming toward the immediate early response by this cluster, allowing for cluster 1 to preferentially upregulate the immediate early response during the dissociation protocol. Cluster 2 exhibited increased transcription of heme oxygenase-1 (*Hmox1*), which is the rate-limiting step in the degradation of heme into iron, biliverdin, and carbon monoxide. Notably, we also observed increased expression of ferroportin (*Slc40a1*), peroxiredoxin (*Prdx1*), ferritin heavy chain (*Fth*), and ferritin light chain (*Ftl*), which supports the supposition that heme/iron handling is an important function of cluster 2 (Figure 2C and Supplemental Figure 3A). Cluster 3 was enriched for genes associated with antimicrobial processes and inflammation, such as *Cxcl2*, macrophage inflammatory protein 1-α (*Ccl3*), and macrophage inflammatory protein 1-β (*Ccl4*), which are involved in the recruitment of inflammatory cells. *CD14*, which is engaged in Toll-like receptor signaling by LPS, was expressed by these cells at high levels (Figure 2C and Supplemental Figure 3B). Cluster 4 expressed transcripts involved in fibrosis, including *Pf4*, *CD206*, and *Stab1* (Supplemental Figure 3C) (14–18). Cluster 5 could be distinguished by the expression of *Vim*, *Ccl2*, *Tnip3*, and *Ccr2* transcripts (Figure 2C and Supplemental Figure 3D). Cluster 6 was enriched in the expression of several genes associated with type I IFN responses, such as *Isg15*, *Ifit3*, and *Ifit2*, indicating a possible role in antiviral responses (Figure 2C and Supplemental Figure 3E). A full listing of the DEGs is provided in the Supplemental Excel File 1.

Gene ontology enrichment analysis (GO: biologic process) determines the extent to which genes associated with specific functions are represented in each cluster. As seen in Table 1, enrichment of specific gene sets could be assigned with varying degrees of confidence to each cluster, with the exception of cluster 5, which contained too few cells in quiescence for a reliable estimation. The most significant terms in clusters 1, 3, and 6 were involved in antibacterial, antiviral, and antifungal responses. Cluster 2 contained terms related to responses to iron, phagocytosis, and wound healing, suggesting involvement in homeostatic functions. Clusters 0 and 4 mapped to few terms, but the analysis contained references to tumor necrosis factor and apoptosis. These disparate gene ontology mappings suggest that each cluster executes a distinct transcriptional program that could be a function of the location in which each cluster resides.

### KRM subpopulations are localized to zones associated with specific nephron structures.

**Figure 3:**
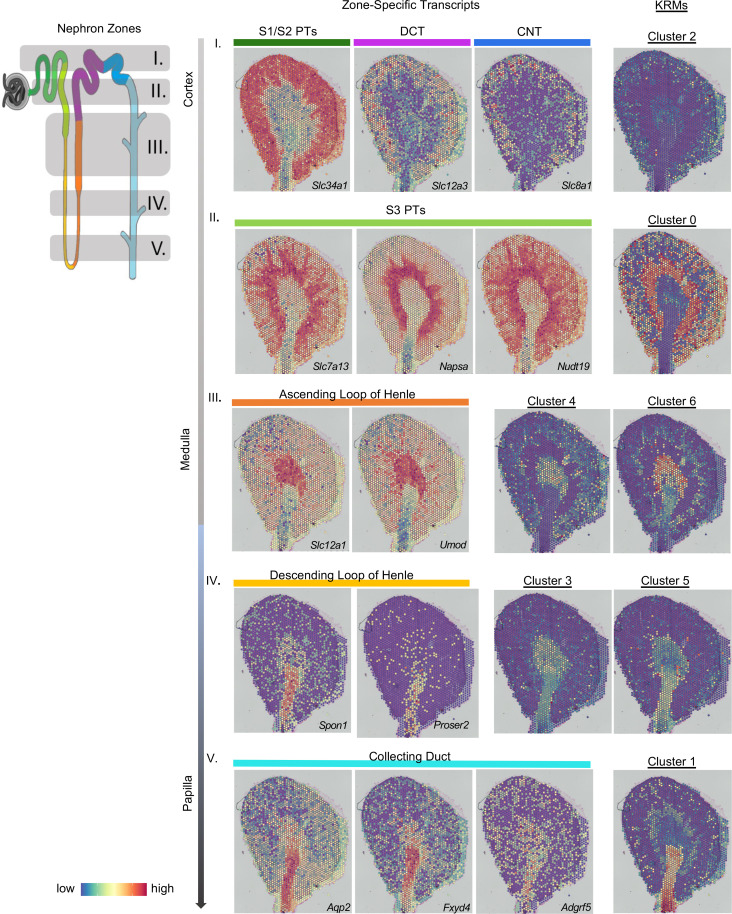
Kidney-resident macrophages are found in distinct regions. An integrated analysis of the single-cell RNA-Seq (scRNA-Seq) and spatial transcriptomics data was performed to localize the kidney-resident macrophage (KRM) clusters on a kidney section. A diagram of a nephron is color coded to delineate different nephron segments (see nephron zones; left). Gray shading and numbering (I–V) describes nephron zones that would be enriched in areas of a kidney cross-section. The spatial location of the nephron segments is shown by mapping segment-specific transcripts onto the histological image (see zone-specific transcripts; middle). Transcript markers are listed in the bottom right-hand corner of each section. Specific nephron segments are listed above each image. Colored bars correspond to the location of the segments from the nephron shown on the left. Row number (I–V) indicates the nephron zone. The integration of the KRM scRNA-Seq data onto the spatial section plots the location of KRM subpopulations within the quiescent kidney (right). The clusters are aligned with the kidney nephron segments that are found in the same zone to highlight the colocalization between KRMs and kidney cells. CNT, connecting tubule; DCT, distal convoluted tubule; PT, proximal tubule.

We identified KRM locations by integrating the scRNA-Seq data (Figure 2A) into spatial transcriptomic data sets generated from kidney sections of 3 individual mice. The spatial matrix was integrated with the scRNA-Seq data set using the anchor-based workflow built into the Seurat package, which allows the probabilistic mapping of scRNA-Seq– and/or single-nucleus RNA-sequencing (snRNA-Seq)–defined clusters onto a histological kidney image (Supplemental Figure 4) (19–22). We validated this approach using a single-nuclear data set from whole kidneys, in which the cell types identified by snRNA-Seq mapped to expected histologic locations in the kidney (Supplemental Figure 5). The 7 transcriptionally defined KRM subpopulations were localized to distinct locations in the kidney (Figure 3, right), corresponding to zones enriched for specific nephron structures (Figure 3, left), which were designated zones I–V and confirmed using zone-specific transcripts (23) (Figure 3, middle). The localization patterns suggest a zonal organization of KRMs from the cortex to the papilla. For example, cluster 0, appearing in zone II, was largely concentrated in the corticomedullary region and appeared to be associated with the S3 segment of the proximal tubule, as determined by marker transcripts for the S3 proximal tubule (*Slc7a13*, *Napsa*, *Nudt19*). Cluster 4 and cluster 6 are predominately located in zone III, and their spatial distributions were strikingly similar to the distributions of genes associated with the ascending loop of Henle. Within zones III and IV, we observed the localization of more than one KRM cluster. In both zones, each cluster was mapped similarly, but with slightly different distributions. The spatial organization of each KRM cluster was unique and associated with specific zones within the kidney (Figure 3).

### Validation of select KRM markers.

**Figure 4:**
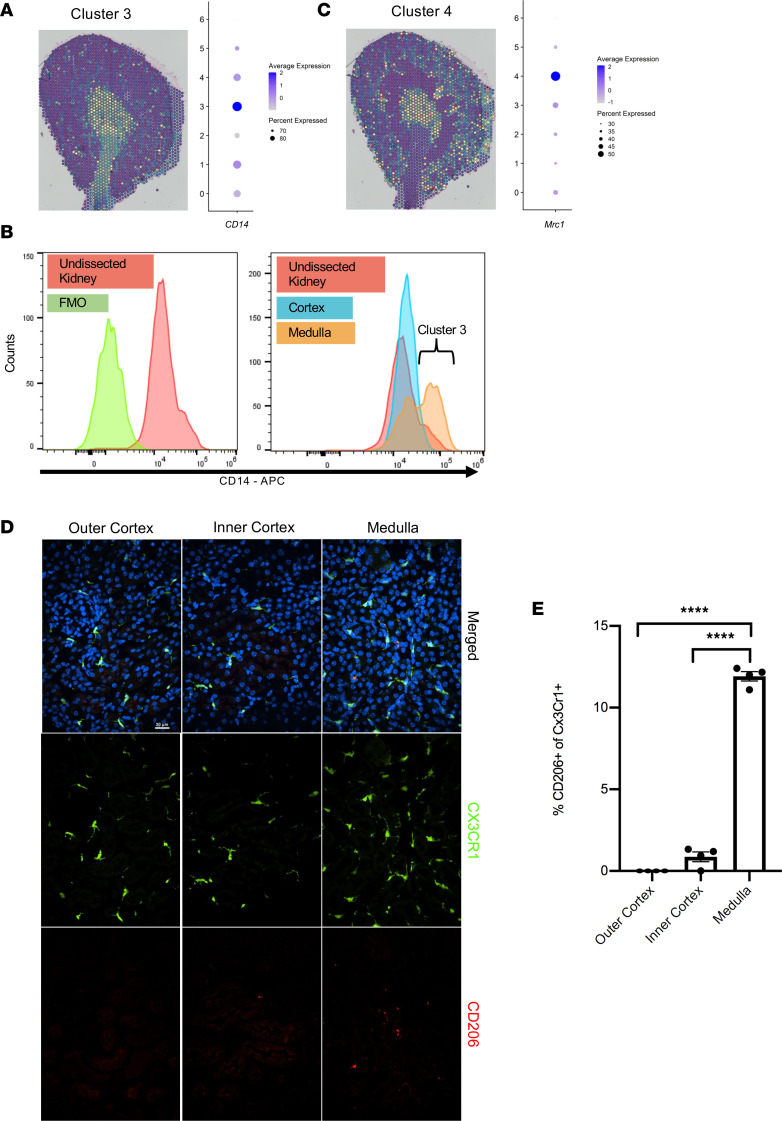
Spatial validation of protein markers. (**A**) Spatial location of cluster 3 overlaid on the histological image and a dot plot of *CD14* transcript expression for each cluster. (**B**) Kidneys were harvested and dissected to separate the cortex from the medulla to confirm the location of cluster 3 CD14^++^ cells in the medulla. Flow cytometry analysis of CD14 expression in KRMs of the whole kidney (left) and dissected cortex compared with medulla (right) along with the fluorescence minus one (FMO) control. (**C**) Spatial location of cluster 4 overlaid on histological image and a dot plot of *Mrc1* transcript expression show that cluster 4 is localized in the outer cortex and inner medulla but not the inner cortex. (**D**) Representative images from immunofluorescence of kidney sections of Cx3Cr1 GFP^+/–^ mice stained with CD206 and the nuclear stain DAPI to validate cluster 4 KRMs by confocal microscopy (original magnification, ×40). Results were averaged from 4 separate fields within each area with 4 mice in total over 2 independent experiments. Scale bar: 20 μm. (**E**) Quantitation from a blinded observer of CD206^+^ KRMs in the outer cortex, inner cortex, and medulla, expressed as a proportion of Cx3Cr1^+^ cells. ****P < 0.0001 by 1-way ANOVA followed by Tukey’s test. Data are shown as mean ± SEM.

To validate the general location of specific clusters shown by spatial transcriptomics, we used flow cytometry to visualize the expression of select proteins that were transcriptionally distinct within clusters. Because cluster 3 resided primarily in the medulla (Figure 4A), we performed flow cytometry to compare expression levels of CD14 protein among cells isolated separately from the medulla and the cortex. Analysis of the most significant DEGs for each KRM cluster by adjusted *P* value indicated that transcripts for CD14, a pathogen-associated molecular pattern receptor involved in the recognition of LPS, were expressed in all KRMs but appeared to substantially increase in cluster 3 (Figure 4A, dot plot). Flow cytometry of kidneys manually dissected into cortex and medulla allowed for the identification of increased CD14^++^ cells among CD45^+^TCRb^–^CD19^–^NK1.1^–^Gr1^–^CD11b^int^F4/80^hi^ KRMs in the medulla but not the cortex of the kidney (Figure 4B). This aligned with the plotting of cluster 3 using spatial transcriptomics, which appeared primarily in the medulla and papilla. In comparison, mannose receptor C-type 1 (*Mrc1*, also known as CD206) expression was increased in cluster 4. This cluster was spatially distributed in the inner medulla and, to a lesser extent, the outer cortex (Figure 4C). Immunofluorescence imaging revealed CD206/Mrc1^+^ KRMs in the outer cortex and inner medulla; however, they were largely absent from the inner cortex, which is consistent with the spatial transcriptomics data (Figure 4, D and E).

### Ischemic injury alters the spatial and transcriptomic profiles of KRM subpopulations, and they remain altered 4 weeks after injury.

**Figure 5:**
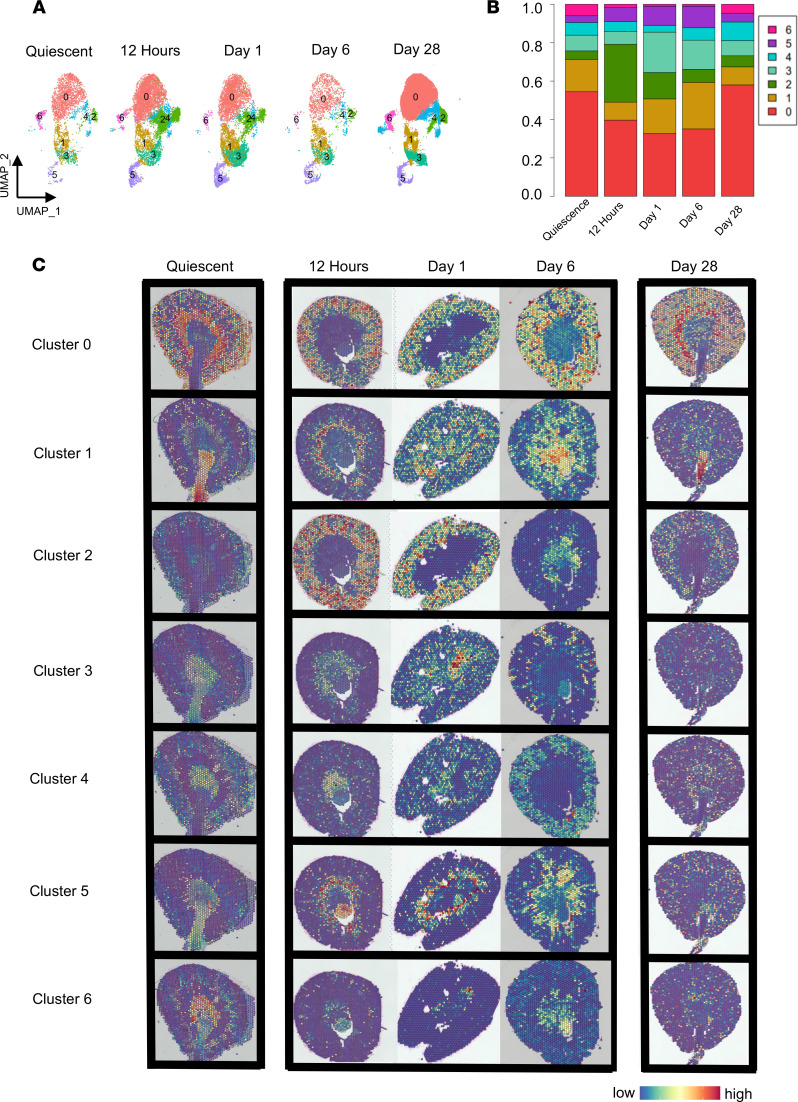
Spatial and proportional changes to KRM subpopulations following injury. (**A**) Uniform manifold approximation and projection (UMAP) plot of KRM clusters at quiescence and 12 hours, day 1, day 6, and day 28 after injury to assess changes after injury. (**B**) Changes in proportions of each cluster over time. (**C**) scRNA-Seq data from each time point integrated with their respective spatial transcriptomic kidney sections to resolve cluster locations. Each row represents a KRM cluster, whereas each column depicts a time point from quiescence to day 28 (left to right). Images were taken with ×4 objective then stitched together so they appear as ×2.

We next determined the effect of ischemic injury on transcriptomic programming and spatial distribution of KRMs as a function of time. Figure 5A shows UMAP plots for KRMs from quiescent kidneys and also those at 12 hours and days 1, 6, and 28 after injury. Relative to quiescent kidneys, proportional changes in the representation of each KRM cluster was observed within 12 hours following the ischemic insult. The proportions at day 28 appear similar to those at quiescence (Figure 5B). Most notable among these acute changes are increases in the proportions of cells in clusters 2 and 5. The GO terms following Days 1 and 6 post-injury are included in Supplemental Tables 1 and 2

Following injury, there was a change in cluster spatial locations (Figure 5C). This was observed in the early inflammatory phase (12 hours and day 1 after injury) as well as in the reparative phase (day 6 after injury). For reference, kidney cell markers at each time point are provided and appear similar to the quiescent profile (Supplemental Figures 6–9). At 12 hours, we identified clusters that appeared to be in intermediate locations between their initial sites and their positions on day 1 and day 6 after injury. This was exemplified by the medullary subpopulations, clusters 1 and 3. During quiescence, these populations were predominantly present in zones V (papilla) and IV (inner medulla), respectively. At 12 hours after injury, cluster 1 and cluster 3 mapped to the cortical/medullary region. By day 1 after insult, cluster 1 was concentrated at the corticomedullary junction but also appeared to be distributed throughout the cortex. Cluster 3, initially found in the papilla, was also found in the cortex (Figure 5C).

Spatial transcriptomics from day 28 shows that some KRM subpopulations did not map to their initial locations (Figure 5C). A comparison of KRM subpopulations at day 28 to their quiescent profile showed that while clusters 0, 1, and 2 mostly mapped to their quiescent location, clusters 3, 4, 5, and 6 appeared scattered throughout the tissue (Figure 5C). Notably, the day 28 kidneys appeared to lose spatially distinct medullary KRM subpopulations.

The GO terms for clusters 1, 3, and 6 at day 28 remained antiviral and antibacterial (Supplemental Table 3). Cluster 2 had reestablished terms involved in iron handling. While GO terms appeared for cluster 0, they had FDRs above the significance level (FDR < 0.05). Clusters 4 and 5 continued to be involved in chemotaxis.

## Discussion

Using scRNA-Seq in conjunction with spatial transcriptomics, we identified, characterized, and localized 7 major subpopulations of KRMs in the mouse kidney before and after ischemic injury. We revealed that KRM subpopulations exist within microenvironmental niches associated with specific zones, roughly approximating layers from the cortex to the papilla. After the injury, the original compartmentalization of KRM subpopulations is lost and is still not fully regained 28 days after injury.

Recent work in the area of the kidney myeloid immune system has focused on increasing the understanding of the various subpopulations in quiescence and following injury (15, 24). Our work expands on these evolving definitions and characterizes protein expression markers for multiple transcriptionally distinct subpopulations. Current studies analyze the macrophage compartment as a whole, which provides important information on infiltrating monocytes but can cloud the understanding of the KRM population. By focusing solely on the KRMs, we have identified at least 7 transcriptionally distinct major subpopulations. Some of these, such as the type I IFN–responding cluster 6, share similarities with resident macrophage populations in other tissues (25, 26). Others, like cluster 5, which expands after ischemic injury, may be unique to the kidney.

In an attempt to generate a comprehensive understanding of kidney macrophages, we identified quiescent protein expression markers for subpopulations that had been identified across injury models previously by other groups. We identified cluster 3 as CD14^++^ and cluster 4 cells as CD206^+^. In human kidneys, CD14^++^ macrophages have been identified as a subpopulation primed against bacterial infection and responsive to salt gradients in models of urinary tract infections (27). Additionally, medullary CD14^++^ cluster 3 KRMs express transcripts involved in neutrophil chemotaxis and activation. Previous studies by Berry et al. demonstrated that medullary CD14^++^ macrophages isolated from human medulla activate neutrophils (27). CD206^+^ macrophages have been identified by other groups in models of unilateral ureteral obstruction and polycystic kidney disease (15, 28). Like in those studies, our CD206^+^ cluster 4 KRMs expressed transcripts for *Stab1*, *Fcrls*, and *Igf1*, which are associated with tissue regeneration and fibrosis. In the liver, resident macrophages that express similar transcripts to cluster 4 are thought to be involved in the prevention of fibrosis following insult (29). Zimmerman et al. identified CD206^+^ KRMs surrounding cysts in polycystic kidney disease development, and inhibition of KRM proliferation decreased CD206^+^ KRM numbers and lessened cyst growth and numbers, suggesting that cluster 4 cells could be involved in cyst formation or fibrosis progression (28).

Stratification of KRMs into specific zones within the kidney was previously unknown. The spatial location of macrophages effects their function in other tissues, such as the lung, spleen, and liver, and response to an immunological challenge, in the case of tuberculosis or tumors (8–12). Although many disease states have known connections with KRMs (30–32) and targeting populations holds great therapeutic promise, successful design and implementation of such strategies are limited by our current understanding of KRM regulation and response to injury. As it relates to location, cluster 0 is initially at the site of the greatest insult during an ischemic event. The lack of oxygen in the tissue damages the mitochondria-heavy S3 proximal tubule cells in the corticomedullary junction (33, 34). Cluster 1 was the second-largest KRM population and was concentrated in the papilla. Its presence in zone V, which has aquaporin 2 gene expression, suggests that cluster 1 cells are found near the collecting ducts. Prior studies have indicated a relationship between macrophages and the collecting duct in the context of IgA nephropathy, with one study suggesting a role in driving fibrosis in that region (35, 36). Cluster 2, which is distinguished by its iron-handling transcripts, was located in the cortex around proximal tubules, where iron is reabsorbed in healthy conditions and accumulates during iron disorders (37). Additionally, the subpopulation locations suggest a coordinated positioning to protect the kidney from infection. The transcriptome and location of clusters 1, 3, and 6 depict a strategic immune barrier from the ureter, the most common origin of kidney infections (38). Macrophage subpopulations in other tissues, such as the lung, gut, and skin, are known to perform this function (39–42). Our work highlights the diversity among the KRMs and the corresponding need to selectively target specific subpopulations with respect to the originating mechanism of insult.

It is similarly important to consider timing, as the KRMs alter their spatial location after injury. This apparent movement can be ascribed either (a) to a physical translocation of the KRMs following injury and/or (b) to KRM subpopulations in specific locations undergoing a transition from transcriptional profiles associated with one cluster to another. The former hypothesis is supported by data showing that 12 hours after injury, clusters 1 and 3 appear at intermediate locations between their quiescent and 24-hour locations. In addition, GO analysis from time points following injury identifies many terms involved in cell migration and motility. This further supports that the KRM subpopulations have location-specific responses to areas of damage within the kidney and may both relocate and alter their transcriptional profile in response to injury. While our data support the possibility that KRMs are migrating, future experiments will need to confirm KRM locomotion.

Nevertheless, the transcriptomic atlas, with many KRM subpopulations no longer expressing their original profiles and existing within new locations, is persistently altered and future therapeutics will need to consider the location at the time of treatment to accurately gain the desired effect. During the course of our studies, the transcriptomic profile failed to return to quiescence, even after 28 days. Given the continued disruption in transcriptional and spatial distribution beyond acute injury, KRMs may influence the transition from AKI to chronic kidney disease (CKD). A single AKI event drastically increases the risk for the development of CKD (43–46), although the mechanisms that underlie the AKI-to-CKD transition remain unclear. Multiple cell types have been implicated, including damaged proximal tubules and vascular cells, in addition to leukocytes (47). KRMs are of particular interest given their capacity for inflammation and fibrosis and continued presence in the tissue beyond an acute injury (48–52). Future studies should consider the effect of the altered KRM subpopulations on long-term kidney function both from native kidneys and following transplant.

This work has characterized the subpopulations of KRMs and determined their location within healthy kidneys. Following insult, we tracked the subpopulations as they appear to relocate throughout the tissue, suggesting locomotion by these cells in response to injury, as a result of tubule cell death and/or transient ischemia to the various subpopulations. Finally, our data confirm a long-hypothesized dysregulation of the immune system following AKI and provide a foundational framework for the increased risk for CKD following an AKI event. The result is a KRM atlas of the murine kidney that can provide a point of reference for future studies into the role of the resident macrophage system in the normal and injured kidney.

## Methods

### Animals.

Male C57BL/6J mice, 12–16 weeks of age, were obtained from The Jackson Laboratory. Mice were housed at the University of Alabama at Birmingham (UAB) animal facilities in compliance with the NIH guidelines regarding the care and use of live animals.

### Bilateral ischemia/reperfusion injury.

Mice were subjected to bilateral ischemia/reperfusion injury, as previously described (3, 4). All surgeries were performed in the morning. Mice were anesthetized using ketamine and xylazine (i.p.). Under aseptic precautions, both kidneys were clamped at the renal pedicle using a microserrefine vascular clamp (Fine Science Tools, 18055-05). After 19 minutes, the clamps were removed to allow reperfusion. Reperfusion was visually confirmed within 1 minute. Body temperature, measured by a rectal thermometer, was carefully maintained at 37°C ± 1°C.

### Flow cytometry/FACS.

KRMs represent approximately 1%–2% of total viable cells in a quiescent kidney. In order to obtain a minimum number of cells to draw meaningful conclusions about KRM subpopulations as determined by scRNA-Seq, we isolated KRMs using flow cytometric sorting. Leukocytes were isolated as previously described (3, 4). Mice were anesthetized under isoflurane and perfused through the left ventricle with 10 mL cold PBS. Kidneys were removed, stripped of the capsule, minced with a razor blade on a glass slide, and placed into Liberase (MilliporeSigma) at 37°C for 30 minutes. The digestion was stopped by adding cold PBS containing 1% BSA, and tissue was further disaggregated through an 18-gauge syringe. Red blood cells were lysed using ACK lysis buffer for 2 minutes at room temperature, and the remaining leukocytes were then washed with ice-cold PBS. Cells were then stained with violet fixable viability dye (Invitrogen L34955) and treated with unlabeled anti-CD16/32 antibody to block Fcγ3 receptors. Cells were subsequently stained using anti–Gr-1 Alexa Fluor 700 (Ly6G, clone 1A8, BioLegend), anti-CD11b super bright 600 (M1/70, Invitrogen), anti-F4/80 APC-eFluor-780 (BM8, Invitrogen), anti-NK1.1 PE-C7 (PK136, Invitrogen), anti-CD45.2 BV-650 (104, BioLegend), anti-MHC II (I-A/I-E) PerCP (M5/114.15.2, BD Biosciences), anti-CD19 super bright 702 (6D5, BioLegend), anti-TCRβ Pe-Cy5 (H57-597, BD Biosciences), and anti-CD14 APC (Sa2-8, Invitrogen) (Supplemental Figure 1).

### Nuclear isolation from whole kidney.

Nuclei were isolated using Nuclei Lysis Buffer containing Nuclei Isolation Kit: Nuclei EZ Prep Buffer (MilliporeSigma) supplemented with cOmplete ULTRA Tablets (MilliporeSigma) and SUPERase IN (Thermo Fisher Scientific) and Promega RNAsin Plus nuclease inhibitors. Kidneys were minced into less than 1 mm pieces in 2 mL Nuclei Lysis Buffer. Samples were transferred to a dounce homogenizer (Kimble) and homogenized. An additional 2 mL Nuclei Lysis Buffer was added to the sample and incubated for 5 minutes on ice. Samples were passed through a 40 μm filter into a 50 mL conical tube. Samples were centrifuged at 500*g* for 5 minutes at 4°C. The supernatant was removed, and the pellet was washed with 4 mL Nuclei Lysis Buffer containing 1% bovine serum albumin for 5 minutes on ice. Samples were centrifuged at 500*g* for 5 minutes at 4°C. Samples were passed through a 5 μm filter into a 50 mL conical tube and then centrifuged again. Nuclei were resuspended in a solution containing PBS, 1% BSA, and 0.1% RNAse inhibitor.

### scRNA-Seq/single-nuclear RNA-Seq.

Purified cells or nuclei were transferred on ice to the UAB Flow Cytometry and Single Cell Core and immediately processed using the Chromium 3′ Single Cell RNA sequencing kit (10× Genomics) according to the manufacturer’s instructions. The cell suspension was counted and combined with a 10× Chromium reagent mixture and loaded into a microfluidic single-cell partitioning device in which lysis and reverse transcriptions occur in microdroplets. The resulting cDNA was amplified by a polymerase chain reaction and subsequently processed to yield bar-coded sequencing libraries. Paired-end sequencing was carried out on an Illumina NovaSeq6000 sequencing platform (Illumina). Reads were processed using the 10× Genomics Cell Ranger Single-Cell Software Suite (version 6.0) on the UAB Cheaha High-Performance Computing Cluster. BCL files were converted to FASTQ files using the CellRanger mkfastq function. CellRanger count was used to align the FASTQ files to the mouse genome (mm10). The gene table, barcode table, and transcriptional expression matrices were created for the analysis indicated below.

### Spatial transcriptomics.

The Visium system relies on a 2-dimensional matrix of 5000 spots distributed on a microscope slide in a 6.5 by 6.5 mm square. Each spot, which contains a poly-dT oligonucleotide with a unique sequence (bar code), is 50 μm in diameter at a distance from the other spots of 100 μm from center to center. Kidneys were embedded in the Optimal Cutting Temperature matrix (Fisher Scientific) and stored at –80°C. Before sectioning, blocks were equilibrated to –10°C for 30 minutes. A 10 μm section was placed onto specialized Spatial Gene Expression slides (10× Genomics) and processed according to the manufacturer’s protocols. Briefly, slides were stained with H&E, and bright-field images were acquired using a Keyence BZ-X700 microscope. Tissues were permeabilized for 18 minutes, and cDNA was generated and used to create second-strand DNA. The resulting cDNA was subject to downstream amplification and library processing for scRNA-Seq. Reads were processed using the 10× Genomics Cell Ranger Single-Cell Software Suite (version 6.0) on the UAB Cheaha High-Performance Computing Cluster. BCL files were converted to FASTQ files using the SpaceRanger mkfastq function. SpaceRanger count was used to align the FASTQ files to the mouse genome (mm10).

### Sequencing analysis.

Both scRNA-Seq and spatial transcriptomics analyses were carried out using packages created for the R statistical analysis environment (version 4.06). Data were primarily analyzed using Seurat (version 3.2.3) and its associated dependencies (19, 53) as previously described (54). Data from each mouse were imported using the Read10X function and then structured into a Seurat object using CreateSeuratObject. For quality control, cells with unique feature counts over 2500 or under 200 were excluded. Data were normalized and scaled using SCTransform (55). Objects from each time point were labeled with unique group IDs and then merged into a single object using the Seurat merge function. Data objects were integrated using the Harmony R package (56). Principal component analysis was performed based on 30 principal components, and then cells were clustered using FindAllMarkers set to a resolution of 0.4. The dimensional reduction was done using UMAP. WebGestaltR was used for gene ontology analysis to identify pathways using the Biologic Process and Kyoto Encyclopedia of Genes and Genomes databases (57).

### Integration of scRNA-Seq and spatial transcriptomics to resolve cell location.

The spatial matrix was integrated with the scRNA-Seq data set using the anchor-based workflow built into the Seurat package. FindTransferAnchors was used on the data object containing all 7 KRM clusters. Using the generated anchor set, the TransferData function created predictions from the KRM reference clusters and applied that to the spatial data set. Using a predictions assay, each subpopulation could be visualized using SpatialFeaturePlots.

### Immunofluorescence staining and imaging.

Kidneys from Cx3Cr1^+^ GFP mice (obtained in-house) were cryosectioned onto glass slides and then fixed in 4% paraformaldehyde for 10 minutes. Tissues were permeabilized using 0.2% Triton X-100 in PBS for 8 minutes, washed 3 times with PBS, and then blocked in a solution containing PBS, 0.1% Triton X-100, 1% bovine serum albumin, and 1% donkey serum for 30 minutes at room temperature. Primary antibodies, including anti-CD206 (abcam, ab64693, 1:250) were diluted in a blocking solution and stained overnight at 4°C. Tissues were washed with PBS; stained with secondary antibodies, including anti-rabbit Alexa Fluor 595 (Invitrogen, A21207, 1:1000) and anti-rat Alexa Fluor 647 (Invitrogen, A21247, 1:1000), for 30 minutes at room temperature; and then washed again with PBS. A 1:1000 DAPI solution was added for 5 minutes at room temperature and then washed with PBS. Slides were mounted with IMMU-MOUNT (Thermo Fisher). All images were captured on a Nikon Spinning-disk confocal microscope with a Yokogawa X1 disk, using a Hamamatsu flash4 sCMOS camera with a ×40 oil immersion objective. Images were processed and analyzed in NIS Elements software (Nikon; version 5.0) and ImageJ (NIH). A blinded observer quantified the number of CD206^+^Cx3cr1^+^ cells of the total Cx3Cr1^+^ cells per high-power field.

### Data availability.

The scRNA-Seq and spatial transcriptomics data generated for this paper were deposited in the NCBI’s Gene Expression Omnibus database (GEO GSE200115).

### Statistics.

GraphPad Prism 8.0 was used for statistical analysis. The mean ± SEM was determined for each treatment group in the experiment. A 1-way ANOVA followed by post hoc analysis was used to determine the statistical significance between groups. *P* values of less than 0.05 were considered significant.

### Study approval.

All animal work performed was reviewed and approved by the Institutional Animal Care and Use Committee at UAB.

## Author contributions

MDC, ENE, JMPL, AA, and JFG designed the experiments. MDC, EE, JMPL, JRL, SL, ZY, and ZL performed the experiments. MDC, EE, KHM, GGR, RK, BKY, AA, and JFG performed data analysis and interpretation. MDC, EE, KHM, AA, and JFG wrote the manuscript. All authors reviewed and approved the manuscript prior to submission. The authorship order among co–first authors was agreed upon after discussion by the authors.

## Supplementary Material

Supplemental data

Supplemental table 1

## Figures and Tables

**Table 1:**
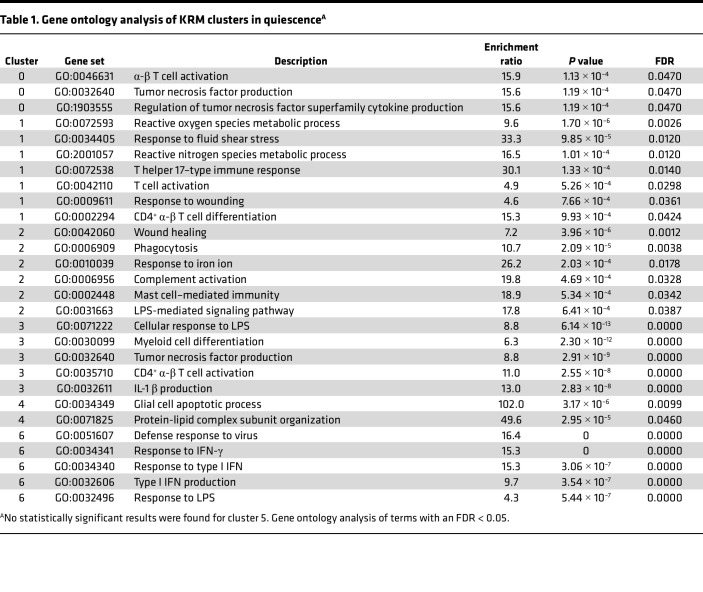
Gene ontology analysis of KRM clusters in quiescence^A^

## Version 1 09/06/2022

In-Press Preview

## Version 2 10/24/2022

Electronic publication
