# Supplementary material for: Resident macrophage subpopulations occupy distinct microenvironments in the kidney
Source: JCI Insight. 2022 Oct 24;7(20):e161078. doi: 10.1172/jci.insight.161078 (PMC9714795; doi:10.1172/jci.insight.161078)
Supplement: Supplemental data [file jciinsight-7-161078-s158.pdf]

<u>Supplemental Materials Table of Contents:</u>

Supplemental Figure 1: Flow cytometry gating scheme to isolate kidney resident macrophages

Supplemental Figure 2: Uniform manifold approximation and projection (UMAP) plot of sequenced kidney resident macrophages (KRMs) from all time points

Supplemental Figure 3: Dot plots of key differentially expressed genes from each cluster

Supplemental Figure 4: Uniform manifold approximation and projection (UMAP) and spatial plots derived from spatial sequencing data

Supplemental Figure 5: Uniform manifold approximation and projection (UMAP) plots of kidney cell types derived from single nuclear RNA sequencing

Supplemental Figure 6: Zone-specific transcripts for spatial transcriptomics section 12 hours after injury

Supplemental Figure 7: Zone-specific transcripts for spatial transcriptomics section 1 day after injury

Supplemental Figure 8: Zone-specific transcripts for spatial transcriptomics section 6 days after injury

Supplemental Figure 9: Zone-specific transcripts for spatial transcriptomics section 28 days after injury

Supplemental Spreadsheet: Full list of differentially expressed genes for all clusters at all time points.

Supplemental Figures:

Supplemental Figure 1

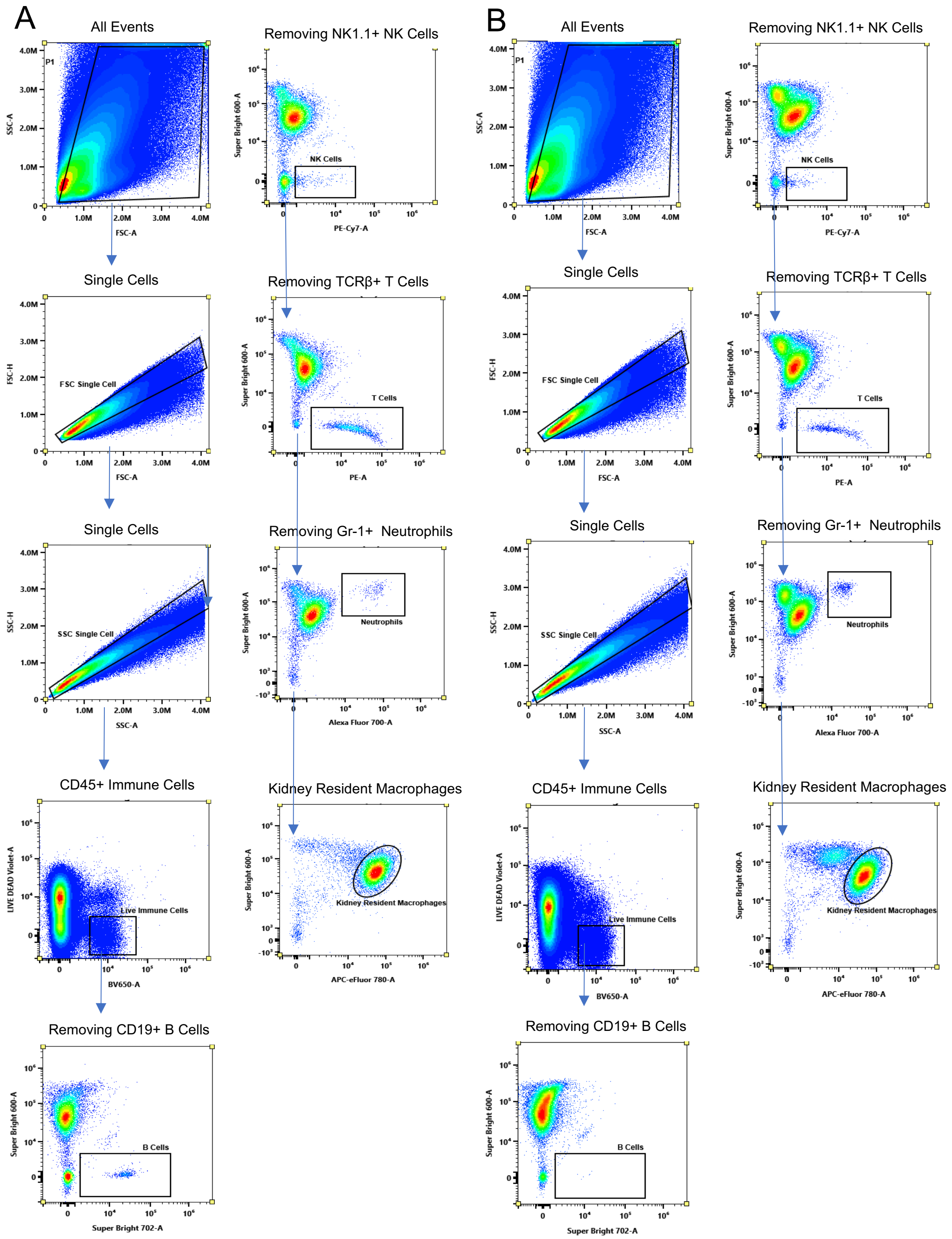

Supplemental Figure 1: Flow cytometry gating scheme to isolate kidney resident macrophages

Gating strategy from kidney single-cell suspensions using pseudocolor defaults both A) pre-injury and B) post-injury.

Supplemental Figure 2

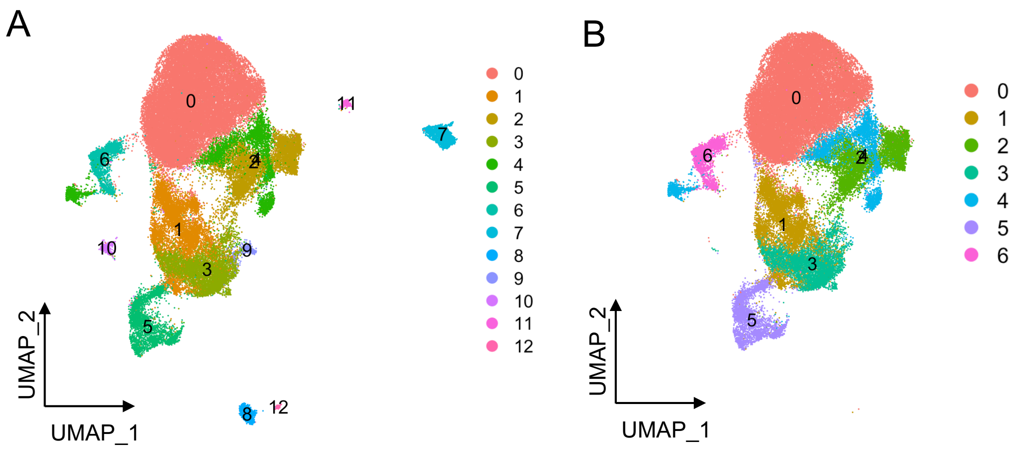

Supplemental Figure 2: Uniform manifold approximation and projection (UMAP) plot of sequenced kidney resident macrophages (KRMs) from all time points

A) Uniform manifold approximation and projection (UMAP) plot of sequenced kidney resident macrophages (KRMs) from all time points (quiescence, 12 hours, day 1, day 6, and day 28 post-injury) demonstrates thirteen unique clusters. Contaminating kidney parenchymal cells and clusters representing less than 1% of the population were included. B) UMAP plot of sequenced KRMs from all time points (quiescence, 12 hours, day 1, day 6, and day 28 post-injury) demonstrates seven unique clusters. Contaminating kidney parenchymal cells and clusters representing less than 1% of the population were omitted from the analysis.

Supplemental Figure 3

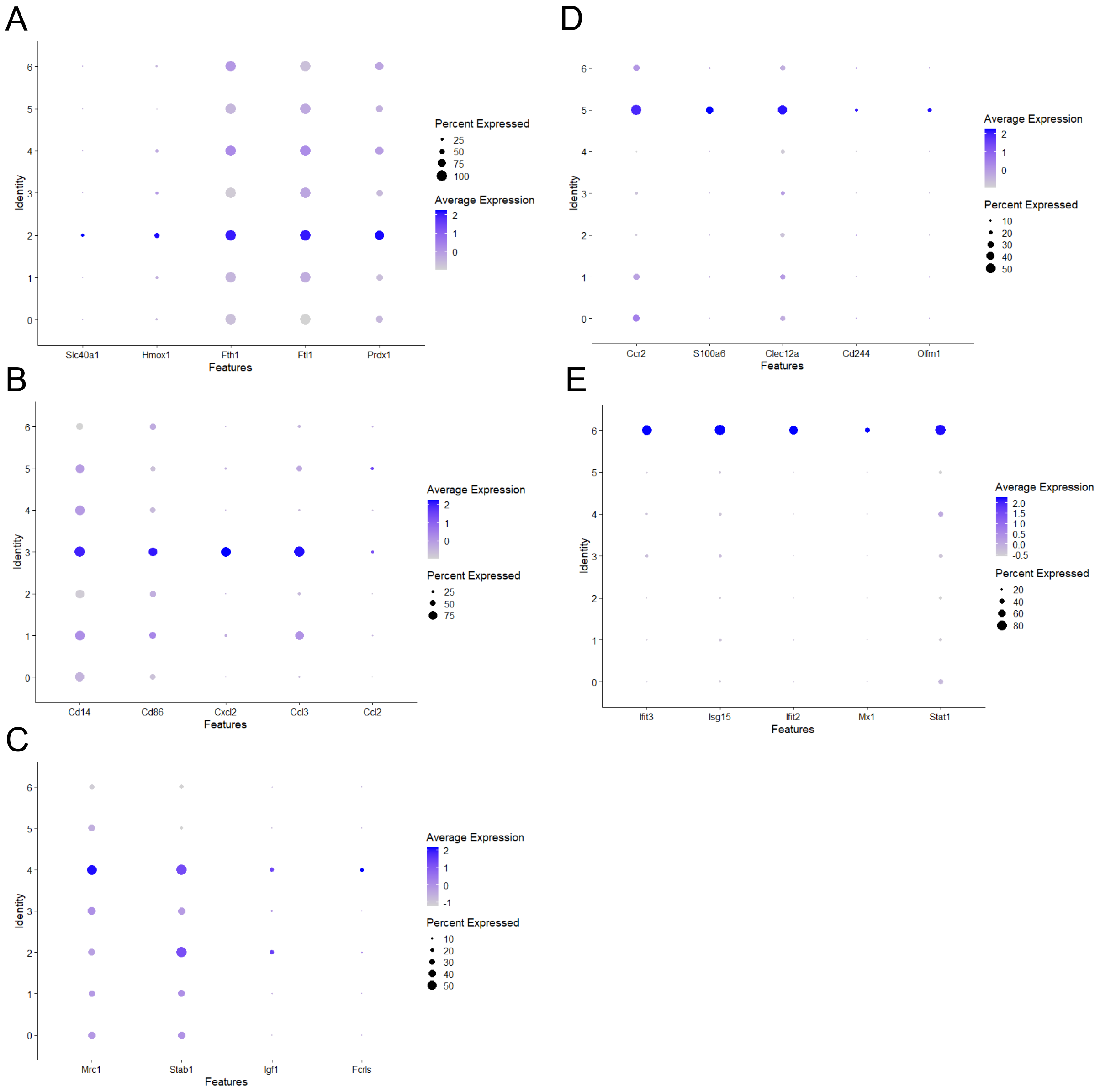

Supplemental Figure 3: Dot plots of key differentially expressed genes from each cluster

Dot plots of key differentially expressed genes from A) cluster 2, B) cluster 3, C) cluster 4, D) cluster 5 and E) cluster 6

Supplemental Figure 4

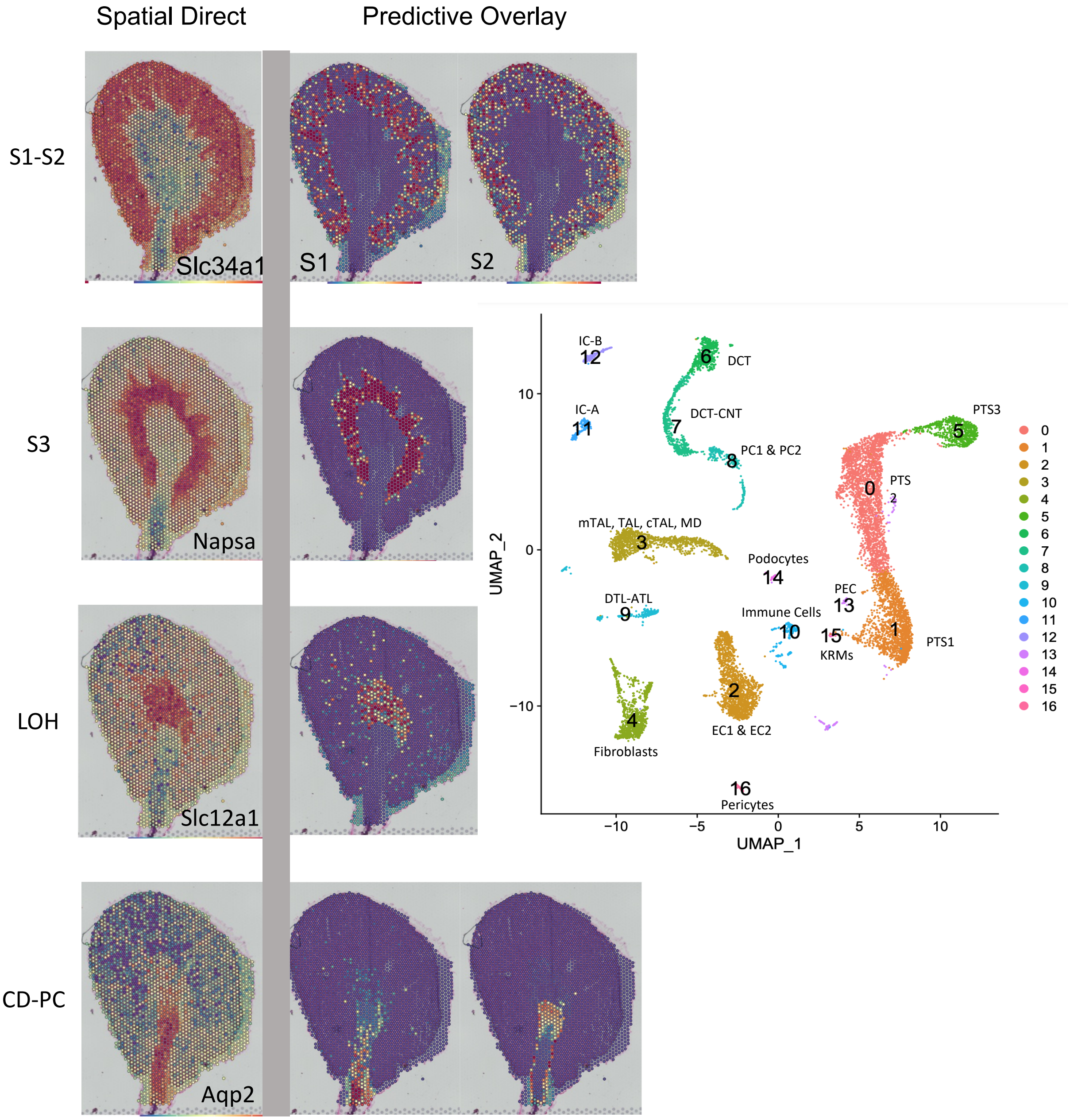

Supplemental Figure 4: Uniform manifold approximation and projection (UMAP) and spatial plots derived from spatial sequencing data

A) Uniform manifold approximation and projection (UMAP) plots derived from spatial sequencing data (left). Spatial plotting of kidney cell types onto spatial histological image (right).

B) Spatial gene expression plots of *Slc34a1 (left)* and *Umod (right)*.

Supplemental Figure 5

A

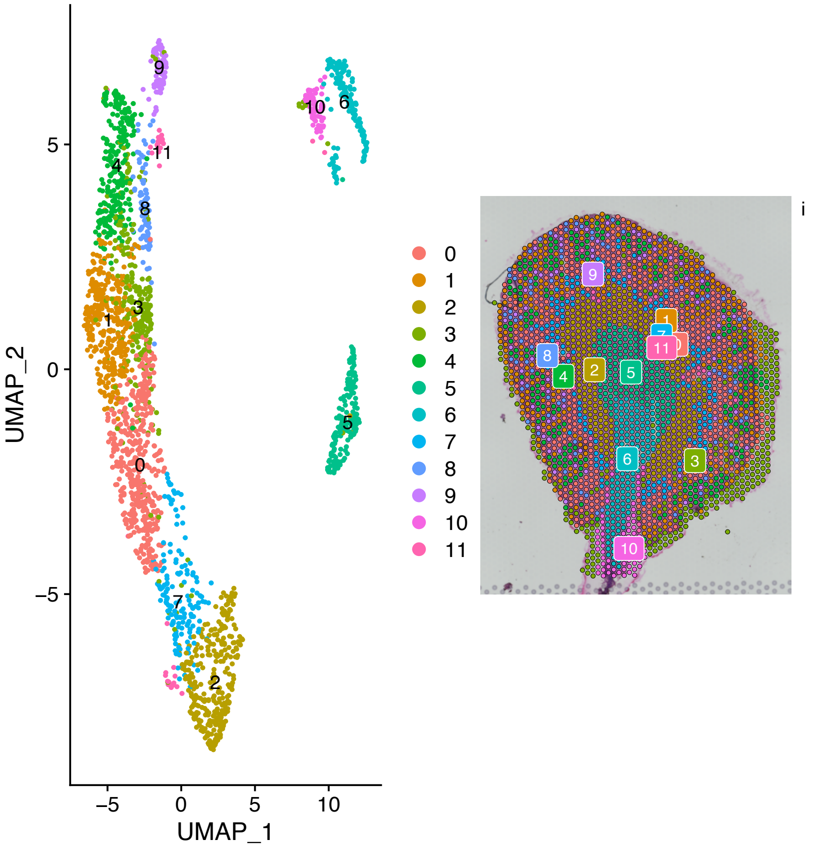

B

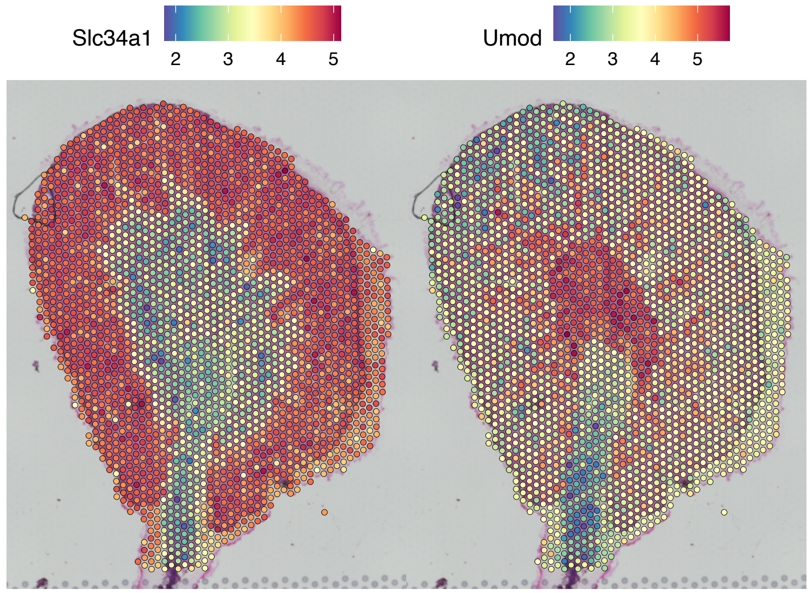

Supplemental Figure 5: Uniform manifold approximation and projection (UMAP) plots of kidney cell types derived from single nuclear RNA sequencing

Uniform manifold approximation and projection (UMAP) plots of kidney cell types derived from single nuclear RNA sequencing (right). Spatial plotting of kidney cell types from single nuclear RNA sequencing onto spatial histological image. The regions of the nephron were mapped using transcripts associated with specific cell types (e.g. Slc34a1 for the S1/S2 region of the proximal tubule or Umod for the ascending loop of Henle).

Supplemental Figure 6 – 12 hour

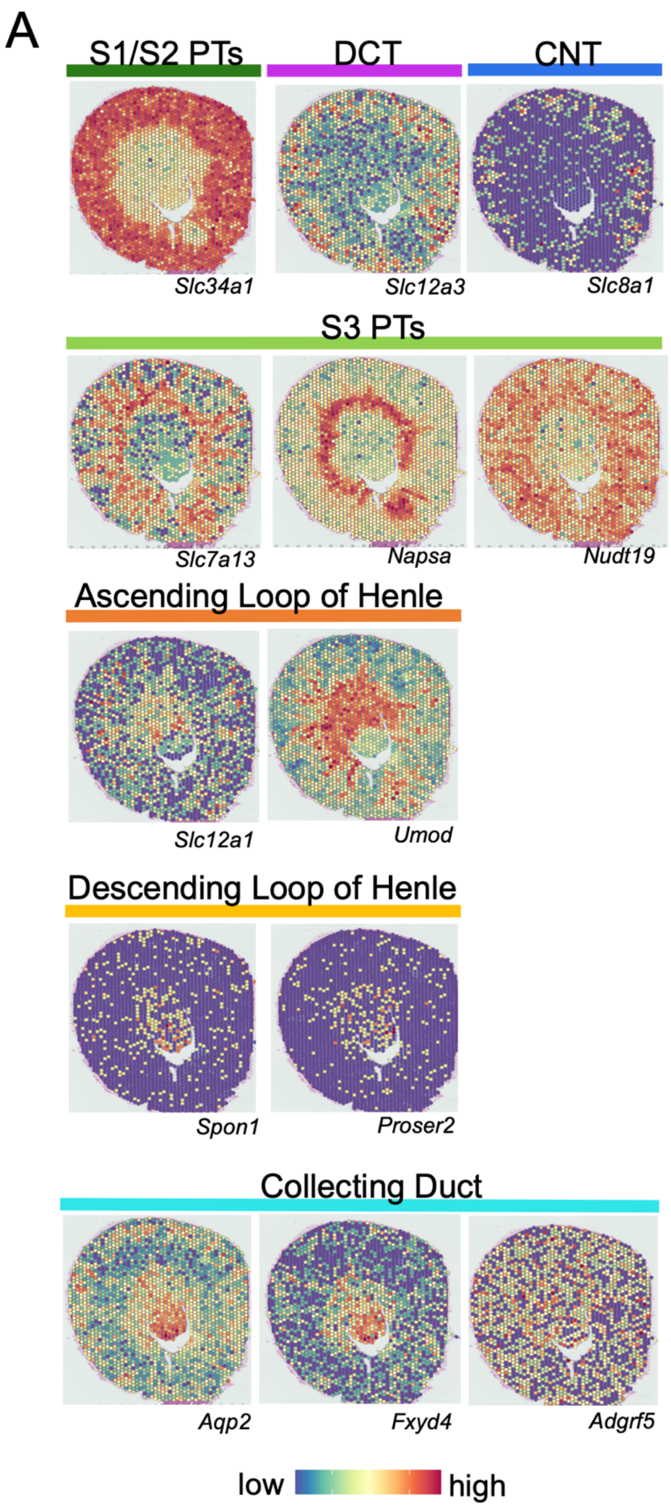

Supplemental Figure 6: Zone-specific transcripts for spatial transcriptomics section 12 hours after injury

The spatial location of the nephron segments are shown by mapping segment-specific transcripts onto the histological image. Transcript markers are listed in the bottom right-hand corner of each section. Specific nephron segments are listed above each image. Colored bars correspond to the location of the segments from the nephron in the left panel from Figure 3.

Supplemental Figure 7 – Day 1

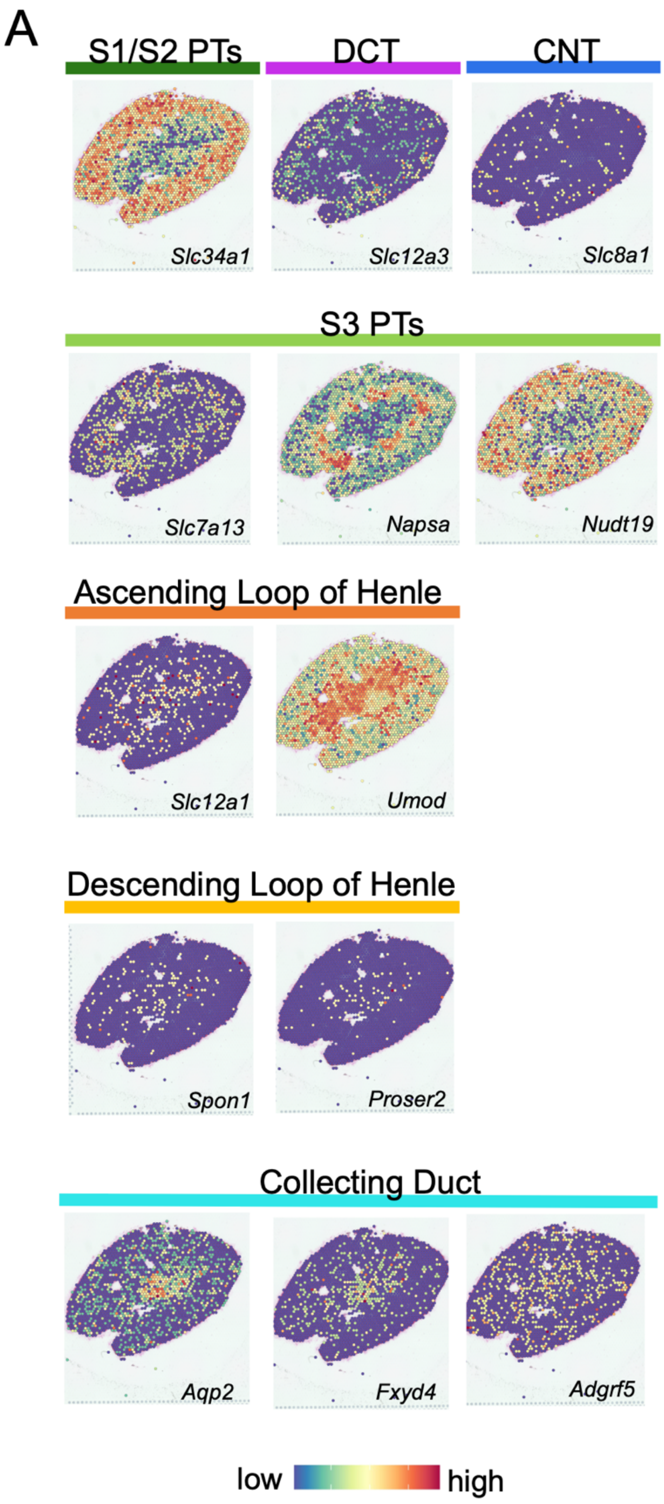

Supplemental Figure 7: Zone-specific transcripts for spatial transcriptomics section 1 day after injury

The spatial location of the nephron segments are shown by mapping segment-specific transcripts onto the histological image. Transcript markers are listed in the bottom right-hand corner of each section. Specific nephron segments are listed above each image. Colored bars correspond to the location of the segments from the nephron in the left panel Figure 3.

Supplemental Figure 8 – Day 6

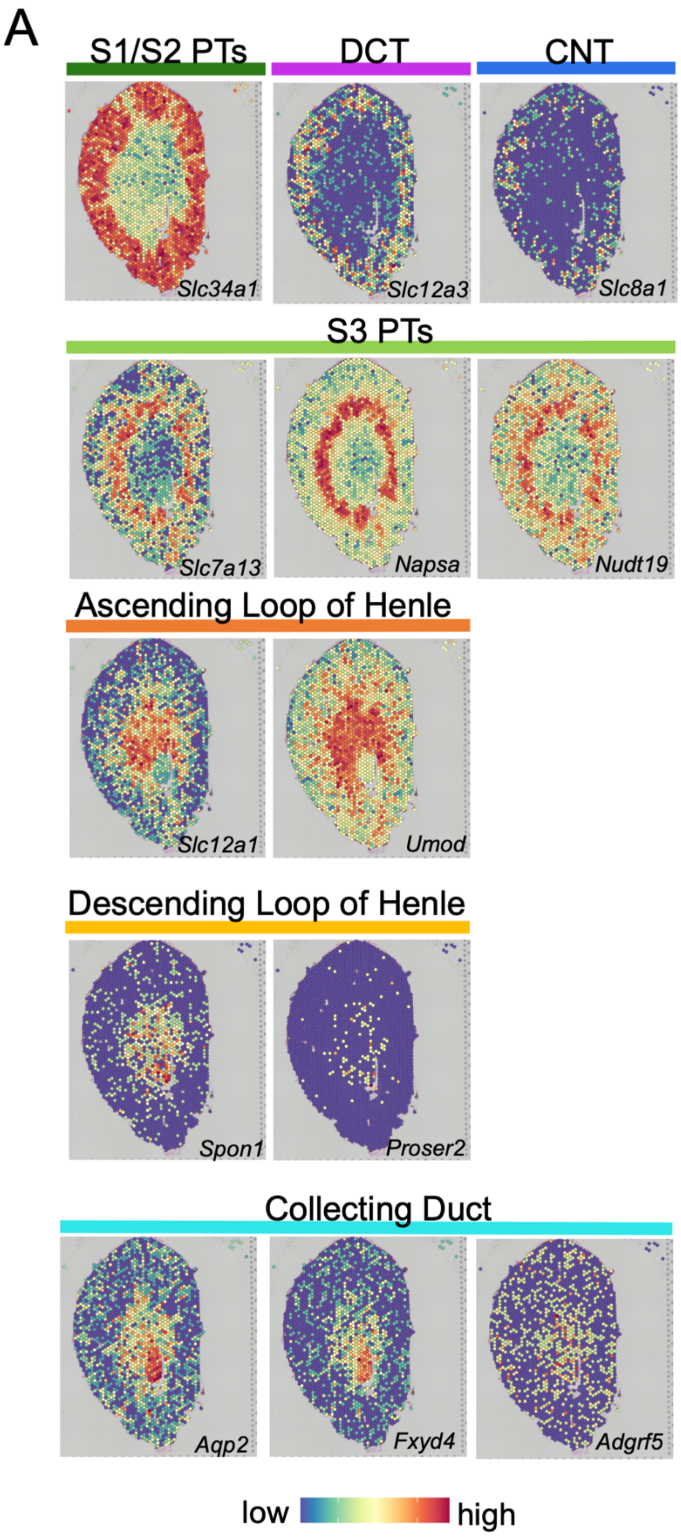

Supplemental Figure 8: Zone-specific transcripts for spatial transcriptomics section 6 days after injury

The spatial location of the nephron segments are shown by mapping segment-specific transcripts onto the histological image. Transcript markers are listed in the bottom right-hand corner of each section. Specific nephron segments are listed above each image. Colored bars correspond to the location of the segments from the nephron in the left panel from Figure 3.

Supplemental Figure 9 – Day 28

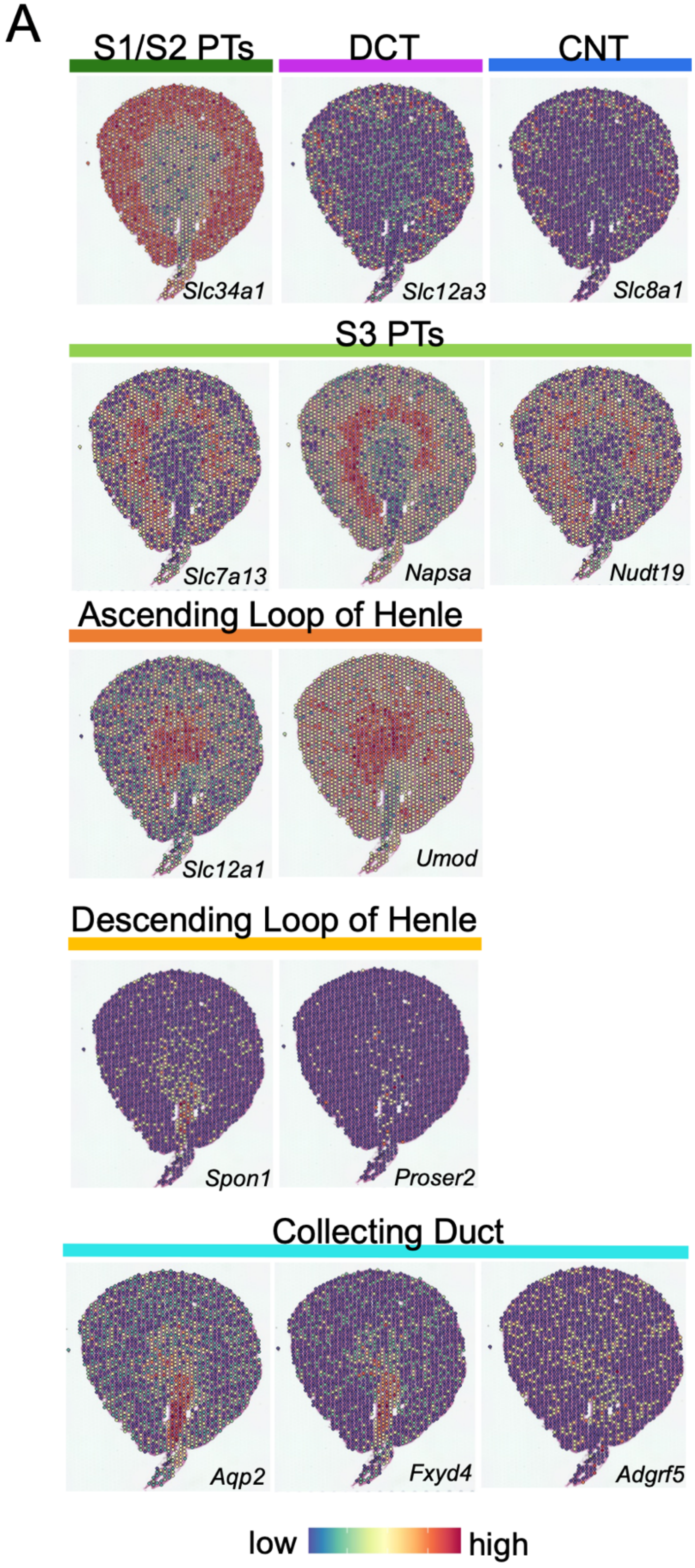

Supplemental Figure 9: Zone-specific transcripts for spatial transcriptomics section 28 days after injury

The spatial location of the nephron segments are shown by mapping segment-specific transcripts onto the histological image. Transcript markers are listed in the bottom right-hand corner of each section. Specific nephron segments are listed above each image. Colored bars correspond to the location of the segments from the nephron in the left panel from Figure 3.

Supplemental
Table 1

Gene Ontology Analysis
Day 1

Cluster	Gene Set	Description	Enrichment Ratio	p-Value	FDR
0	GO:0002478	antigen processing and presentation of exogenous peptide antigen	105.4	1.60E-04	0.0504
0	GO:0033077	T cell differentiation in thymus	32.7	1.00E-04	0.0504
0	GO:0019724	B cell mediated immunity	25.9	2.01E-04	0.0504
0	GO:2001233	regulation of apoptotic signaling pathway	12.1	4.35E-05	0.0504
1	GO:0043921	modulation by host of viral transcription	81.3	6.48E-06	0.0009
1	GO:0002548	monocyte chemotaxis	39.9	5.72E-05	0.0047
1	GO:0032611	interleukin-1 beta production	29.5	1.41E-04	0.0090
1	GO:0002688	regulation of leukocyte chemotaxis	24.9	1.90E-05	0.0021
1	GO:0007088	regulation of mitotic nuclear division	16.5	9.37E-05	0.0069
1	GO:0032496	response to lipopolysaccharide	16.0	2.18E-08	0.0000
1	GO:1903706	regulation of hemopoiesis	14.4	4.85E-08	0.0000
1	GO:0050727	regulation of inflammatory response	13.0	5.33E-06	0.0008
1	GO:0030099	myeloid cell differentiation	11.1	1.28E-05	0.0015
2	GO:0034368	protein-lipid complex remodeling	95.1	1.97E-04	0.0614
2	GO:0033006	regulation of mast cell activation involved in immune response	62.4	4.63E-04	0.0904
2	GO:0010039	response to iron ion	58.7	5.23E-04	0.0904
3	GO:1990869	cellular response to chemokine	23.2	1.90E-09	0.0000
3	GO:0045428	regulation of nitric oxide biosynthetic process	22.7	2.50E-07	0.0000
3	GO:0032611	interleukin-1 beta production	21.4	3.59E-07	0.0000
3	GO:0030593	neutrophil chemotaxis	18.5	9.83E-08	0.0000
3	GO:1990266	neutrophil migration	15.7	3.13E-07	0.0000
3	GO:0071222	cellular response to lipopolysaccharide	14.8	3.63E-12	0.0000
3	GO:0032640	tumor necrosis factor production	13.4	1.44E-07	0.0000
3	GO:0071248	cellular response to metal ion	12.3	4.95E-08	0.0000
3	GO:2000377	regulation of reactive oxygen species metabolic process	11.8	6.86E-08	0.0000
3	GO:0060326	cell chemotaxis	8.8	1.92E-07	0.0000
3	GO:0097193	intrinsic apoptotic signaling pathway	8.7	2.18E-07	0.0000
3	GO:0050900	leukocyte migration	8.6	5.59E-08	0.0000
3	GO:0042110	T cell activation	8.1	3.93E-10	0.0000
4	GO:1990868	response to chemokine	35.5	9.43E-10	0.0000
4	GO:0097529	myeloid leukocyte migration	20.8	3.81E-10	0.0000
4	GO:0070555	response to interleukin-1	14.7	1.54E-04	0.0223
4	GO:0050920	regulation of chemotaxis	10.7	9.85E-05	0.0156
5	GO:0097529	myeloid leukocyte migration	8.2	4.61E-10	0.0000
5	GO:0030595	leukocyte chemotaxis	8.2	1.23E-10	0.0000
5	GO:0006909	phagocytosis	8.0	9.97E-09	0.0000
5	GO:0042110	T cell activation	3.8	2.65E-06	0.0002
6	GO:0035457	cellular response to interferon-alpha	97.3	1.61E-11	0.0000
6	GO:0034340	response to type I interferon	27.0	1.09E-06	0.0001
6	GO:0009615	response to virus	20.6	0	0.0000
6	GO:0045088	regulation of innate immune response	19.7	0	0.0000
6	GO:0034341	response to interferon-gamma	18.9	1.60E-12	0.0000

Supplemental Table 1: Gene ontology analysis of KRM clusters 1 day after injury

Gene ontology analysis of terms with an FDR < 0.05, with the exceptions of clusters 0 and 2 where no gene sets met this requirement. The top four and three terms were listed, respectively.

Supplemental Table 2

Gene Ontology Analysis
Day 6[1]

Cluster	Gene Set	Description	Enrichment Ratio	p-Value	FDR
1	GO:0038066	p38MAPK cascade	55.6	7.56E-07	0.0003
1	GO:0030099	myeloid cell differentiation	11.7	1.63E-06	0.0004
1	GO:0070371	ERK1 and ERK2 cascade	10.7	1.68E-05	0.0022
1	GO:0071222	cellular response to lipopolysaccharide	14.2	2.44E-05	0.0028
1	GO:0097191	extrinsic apoptotic signaling pathway	14.1	2.49E-05	0.0028
1	GO:0042110	T cell activation	8.0	8.44E-05	0.0070
1	GO:0002685	regulation of leukocyte migration	13.1	2.33E-04	0.0145
2	GO:0030595	leukocyte chemotaxis	10.4	4.86E-06	0.0050
2	GO:0070371	ERK1 and ERK2 cascade	6.9	1.93E-05	0.0080
2	GO:0097530	granulocyte migration	10.9	9.54E-05	0.0138
2	GO:1990868	response to chemokine	14.0	1.94E-04	0.0208
2	GO:0072676	lymphocyte migration	12.6	2.86E-04	0.0254
2	GO:0043542	endothelial cell migration	8.0	4.10E-04	0.0297
2	GO:0002548	monocyte chemotaxis	17.4	6.79E-04	0.0428
3	GO:0032496	response to lipopolysaccharide	14.9	0	0.0000
3	GO:1903706	regulation of hemopoiesis	11.5	1.35E-14	0.0000
3	GO:0002685	regulation of leukocyte migration	14.1	3.20E-10	0.0000
3	GO:0034612	response to tumor necrosis factor	13.8	4.24E-10	0.0000
3	GO:1990266	neutrophil migration	19.7	7.79E-10	0.0000
3	GO:0097191	extrinsic apoptotic signaling pathway	12.2	1.55E-09	0.0000
3	GO:1990868	response to chemokine	22.6	2.33E-09	0.0000
3	GO:0097530	granulocyte migration	15.9	5.15E-09	0.0000
3	GO:0070372	regulation of ERK1 and ERK2 cascade	8.8	1.02E-08	0.0000
3	GO:0030595	leukocyte chemotaxis	12.0	1.04E-08	0.0000
3	GO:0002573	myeloid leukocyte differentiation	11.5	1.51E-08	0.0000
3	GO:0071621	granulocyte chemotaxis	16.7	2.60E-08	0.0000
3	GO:0070555	response to interleukin-1	16.4	2.97E-08	0.0000
3	GO:0002224	toll-like receptor signaling pathway	18.3	1.09E-07	0.0000
3	GO:0010574	regulation of vascular endothelial growth factor production	38.7	1.72E-07	0.0000
3	GO:0032640	tumor necrosis factor production	13.1	1.76E-07	0.0000
4	GO:0097529	myeloid leukocyte migration	19.1	9.17E-11	0.0000
4	GO:0071674	mononuclear cell migration	33.9	1.37E-09	0.0000
4	GO:1990868	response to chemokine	29.5	3.67E-09	0.0000
4	GO:0070555	response to interleukin-1	21.4	3.45E-08	0.0000
4	GO:0034612	response to tumor necrosis factor	13.1	1.01E-06	0.0003
4	GO:0046718	viral entry into host cell	27.1	1.10E-06	0.0003
4	GO:0070371	ERK1 and ERK2 cascade	8.3	4.59E-06	0.0009
4	GO:0002685	regulation of leukocyte migration	11.5	1.32E-05	0.0023
4	GO:0050920	regulation of chemotaxis	10.7	1.99E-05	0.0033
4	GO:0035455	response to interferon-alpha	44.8	4.00E-05	0.0059
5	GO:0002548	monocyte chemotaxis	81.4	2.11E-12	0.0000
5	GO:0048247	lymphocyte chemotaxis	58.1	2.19E-08	0.0000
5	GO:0048245	eosinophil chemotaxis	118.6	3.17E-08	0.0000
5	GO:0071621	granulocyte chemotaxis	30.9	3.46E-08	0.0000
5	GO:0009611	response to wounding	10.9	7.58E-08	0.0000
5	GO:0030593	neutrophil chemotaxis	31.9	4.60E-07	0.0001
5	GO:0070371	ERK1 and ERK2 cascade	12.1	1.35E-06	0.0003
5	GO:0050863	regulation of T cell activation	12.3	7.70E-06	0.0011
5	GO:0090025	regulation of monocyte chemotaxis	71.2	9.77E-06	0.0014
5	GO:0071675	regulation of mononuclear cell migration	39.5	5.89E-05	0.0060
5	GO:0045625	regulation of T-helper 1 cell differentiation	98.8	1.80E-04	0.0127
5	GO:0032717	negative regulation of interleukin-8 production	79.1	2.85E-04	0.0178
6	GO:0035456	response to interferon-beta	165.0	0	0.0000
6	GO:0009615	response to virus	34.6	1.11E-16	0.0000
6	GO:0002218	activation of innate immune response	41.8	8.53E-12	0.0000
6	GO:0035455	response to interferon-alpha	137.5	1.72E-08	0.0000
6	GO:0034340	response to type I interferon	63.5	1.38E-05	0.0072

[1] No significant results were returned for cluster 0

Supplemental Table 2: Gene ontology analysis of KRM clusters 6 days after injury

Gene ontology analysis of terms with an FDR < 0.05.

Supplemental Table 3

Gene Ontology Analysis
Day 28[1]

Cluster	Gene Set	Description	Enrichment Ratio	p-Value	FDR
1	GO:0032496	response to lipopolysaccharide	16.6	1.58E-08	0.0000
1	GO:0043921	modulation by host of viral transcription	84.3	5.79E-06	0.0008
1	GO:0070371	ERK1 and ERK2 cascade	12.3	7.17E-06	0.0008
1	GO:0051403	stress-activated MAPK cascade	14.4	2.16E-05	0.0018
1	GO:0050729	positive regulation of inflammatory response	23.2	2.47E-05	0.0020
1	GO:0002548	monocyte chemotaxis	41.3	5.11E-05	0.0036
1	GO:0051384	response to glucocorticoid	17.5	7.53E-05	0.0049
2	GO:0010631	epithelial cell migration	18.7	5.26E-06	0.0096
2	GO:0071281	cellular response to iron ion	166.4	6.23E-05	0.0248
2	GO:0034368	protein-lipid complex remodeling	95.1	1.97E-04	0.0455
2	GO:0001936	regulation of endothelial cell proliferation	25.4	2.11E-04	0.0470
3	GO:0032496	response to lipopolysaccharide	16.9	6.66E-16	0.0000
3	GO:0071219	cellular response to molecule of bacterial origin	19.3	9.01E-13	0.0000
3	GO:0051384	response to glucocorticoid	17.8	1.45E-08	0.0000
3	GO:0050729	positive regulation of inflammatory response	20.7	4.35E-08	0.0000
3	GO:0070098	chemokine-mediated signaling pathway	28.6	6.16E-08	0.0000
3	GO:0002548	monocyte chemotaxis	35.1	2.99E-07	0.0000
3	GO:0070371	ERK1 and ERK2 cascade	9.4	4.08E-07	0.0000
3	GO:0050766	positive regulation of phagocytosis	32.5	4.39E-07	0.0000
3	GO:0008630	intrinsic apoptotic signaling pathway in response to DNA damage	19.7	5.77E-07	0.0000
3	GO:0002685	regulation of leukocyte migration	13.4	8.48E-07	0.0001
3	GO:1902532	negative regulation of intracellular signal transduction	7.3	9.00E-07	0.0001
3	GO:2001233	regulation of apoptotic signaling pathway	8.2	1.22E-06	0.0001
4	GO:0070098	chemokine-mediated signaling pathway	63.2	3.95E-07	0.0014
4	GO:0034341	response to interferon-gamma	35.4	4.05E-06	0.0050
4	GO:0071219	cellular response to molecule of bacterial origin	21.3	3.03E-05	0.0235
4	GO:0034369	plasma lipoprotein particle remodeling	112.9	1.39E-04	0.0404
4	GO:0032496	response to lipopolysaccharide	14.0	1.54E-04	0.0404
4	GO:0070371	ERK1 and ERK2 cascade	13.8	1.61E-04	0.0404
4	GO:0097530	granulocyte migration	26.2	1.88E-04	0.0404
5	GO:0034341	response to interferon-gamma	13.9	1.76E-08	0.0001
5	GO:0048247	lymphocyte chemotaxis	20.2	4.73E-06	0.0045
5	GO:0070098	chemokine-mediated signaling pathway	13.7	3.17E-05	0.0164
5	GO:0006909	phagocytosis	7.4	1.62E-04	0.0481
6	GO:0045089	positive regulation of innate immune response	21.9	0	0.0000
6	GO:0045071	negative regulation of viral genome replication	43.8	4.56E-13	0.0000
6	GO:0071346	cellular response to interferon-gamma	19.2	9.55E-10	0.0000
6	GO:1900225	regulation of NLRP3 inflammasome complex assembly	56.1	1.86E-05	0.0019
6	GO:0038094	Fc-gamma receptor signaling pathway	56.1	1.86E-05	0.0019
6	GO:0001961	positive regulation of cytokine-mediated signaling pathway	24.3	2.16E-05	0.0022
6	GO:0002757	immune response-activating signal transduction	6.6	2.77E-05	0.0027
6	GO:0032680	regulation of tumor necrosis factor production	10.1	2.93E-05	0.0029
6	GO:0032481	positive regulation of type I interferon production	20.3	4.48E-05	0.0037
6	GO:0032648	regulation of interferon-beta production	19.9	4.86E-05	0.0040
6	GO:0034121	regulation of toll-like receptor signaling pathway	17.4	8.25E-05	0.0060
6	GO:0032647	regulation of interferon-alpha production	28.1	1.62E-04	0.0101

[1] No significant results were returned for cluster 0

Supplemental Table 3: Gene ontology analysis of KRM clusters 28 days after injury

Gene ontology analysis of terms with an FDR < 0.05.

Supplemental Spreadsheet: Full list of differentially expressed genes for all clusters at all time points.
